# Device-related patient outcomes for coronary stents: A MAUDE database analysis

**DOI:** 10.1016/j.heliyon.2024.e40908

**Published:** 2024-12-04

**Authors:** Zihan Gao, Willie Lei, Eleanor Gao, Sujata Bhatia

**Affiliations:** a David Geffen School of Medicine, University of California, Los Angeles, CA, United States; b University of Waterloo, Waterloo, ON, Canada; c University of Toronto, Toronto, ON, Canada; d Harvard University, Cambridge, MA, United States

**Keywords:** Coronary stents, MAUDE database, Population trends

## Abstract

The growing prevalence of coronary artery diseases in the US corresponds to the increasing use of minimally invasive techniques that require coronary stents. Although extensive research is available on the perioperative outcomes of the 3 stent options – bare-metal stents (BMS), drug-eluting stents (DES), and bioresorbable drug-eluting stents (BVS), a knowledge gap exists in the longitudinal monitoring of patient outcomes due to device-related causes. Therefore, our study examines the device-related patient outcome and the relative performance for BMS, DES, and BVS. Data on 3 device outcomes (deaths, injuries, and malfunction) for each stent type was obtained from the January 2011 to February 2020 Manufacturer and User Facility Device Experience (MAUDE) database. Statistical visualizations and analysis were used to identify trends and significant differences between groups. Of a total of 68,618 adverse event reports, DES, BMS, and BVS each accounted for 88.5 %, 10.2 %, and 1.25 % of the cases, respectively. Device malfunctions were the most reported event (47.2 %), followed by injuries (44.1 %) and deaths (8.66 %). Over time, BMS malfunction rates showed a steady decrease (R = −0.87), while DES malfunction rates increased significantly (R = 0.79). An inversely proportional relationship between DES injuries and malfunctions was observed. The increase in DES malfunctions was 4 times greater than the decrease in BMS malfunctions. Approximately 7 % of reported adverse events were classified as misreported, with most involving DES. These results suggest 2 plausible interpretations: 1) reporting categorization for devices shifted from injuries to malfunction, and 2) stents choice is transitioning from BMS to DES. Our findings also highlight the need to improve reporting accuracy for MAUDE database data.

## 1 Introduction

Over 18.2 million adults in the US have coronary artery diseases [1], corresponding intervention are in high demand, one of the most common being angioplasty procedures. With over 965,000 angioplasty procedures performed annually in the US [2], patient clinical outcome after the minimally invasive procedure for the different available coronary stent types is a critical assessment to allow for optimal treatment decision. Currently on the market are 3 main types of coronary stents: 1) bare-metal stent (BMS), 2) drug-eluting stent (DES), and 3) bioresorbable drug-eluting stent (BVS) [3]. A voluminous amount of studies on coronary stents have suggested better outcomes after DES, including lower mortality, risk of revascularization, and stent thrombosis and restenosis [[4], [5], [6], [7]]. However, a comparison between BMS and DES showed that BMS had superior mechanical performance in several parameters than DES, suggesting room for improvement for the design of future coronary stents [8]. Furthermore, more recent research on the emerging BVS have demonstrated advantages over DES such as restoring normal vasomotion and endothelial function [9,10]. However, though extensive research has been directed to understand the perioperative outcomes of angioplasties using different types of stents, the comparison of longitudinal device-related trends and outcomes for each stent remains limited. With the different stent options available and a need to extrapolate the associated clinical outcomes to make informed clinical decisions, this study aims to examine the device-related patient outcome for 3 main types of coronary stents and their relative performance of these devices through a longitudinal analysis of available data.

## 2 Methods

Data used in this study were obtained from the U.S. Food and Drug Administration (FDA) Manufacturer and User Facility Device Experience (MAUDE) database. This database contains all adverse events reported by mandatory reporters (manufacturers, importers, and device user facilities) and voluntary reporters (healthcare professionals, consumers, and patients) [11]. To investigate the longitudinal changes in device outcomes, a decade of longitudinal data January 2011 to February 2020 were used, eliminating any trend deviation caused by the pandemic.

FDA product codes were used to identify the 3 categories of coronary stents: BMS (MAF), DES (NIQ), and BVS (PNY) [12]. Medical device outcome records were grouped based on the device outcome with the options of unknown/missing, death, injury, malfunction, and other labeled as ∗, D, I, M, and O, respectively. Device outcomes including death, injury, and malfunction were interpreted according to the MAUDE database definitions. Specially, the “death” status is assigned as the device-related outcome when a device may have caused or contributed to a death of a patient or if the clinical cause of death is unknown [13,14]. The “injury” outcome indicates a life-threatening adverse event causing permanent impairment of bodily structure or function, or requires medical or surgical intervention to prevent such outcomes. The “malfunction” device outcome indicates a failure of a device to meet performance specifications [13]. Device outcomes with the status of unknown/missing and others were excluded from the data analysis due to small sample size. Furthermore, all records with duplicated information or had missing key information from the necessary columns were excluded.

Descriptive statistics such as the percentages of all devices were collected from the data sample. Trends for each device outcomes were mapped using scatter plot graphs for each of the product codes. Linear regressions were performed on all graphs that showed visible trends in the mapped data. To minimize the effect of reporting time, which varies based on the companies, the event date was used instead of the report date, which is the default used to categorize the data files in the MAUDE database. Lastly, misreporting rates were identified for BMS and DES. The misreporting rate is defined as the number of deaths found by searching key terms (Table 1) in the information column reported in malfunction and injuries and dividing the tallied value by the total number of deaths found in all device outcomes.

**Table 1:** Search terms applied to FDA MAUDE event narratives to detect misreported deaths.

Patient expired
Patient died
Subsequently expired
Decedent
Time of death
Patients died
Patient later expired

All data analyses were performed using Python 3.10.4, PostgreSQL, and Statistical Package for the Social Sciences (SPSS). This study was exempted from the institutional ethical review given the analysis of data accessible from a public domain.

## 3 Results

A total of 68,618 records of devices associated with unique adverse event reports were found. Of all adverse event records, majority were DES (88.5 %), followed by BMS (10.2 %) and BVS (1.3 %). In addition, based on device outcome, most reported were malfunctions (47.2 %) followed closely by injury (44.1 %), and lastly death (8.7 %) (Table 2).

**Table 2:** Reported adverse events for stents from January 2011 to March 2020 in FDA MAUDE Prevalence divided into the product code and device outcome.

	Malfunction	Injury	Death	Total
BMS	3,182	3,144	676	7,002
DES	2,9176	26,409	5,172	60,757
BVS	31	732	96	859
Total	32,389	30,285	5,944	**68618**

∗ BMS bare metal stents; DES drug eluting stents; BVS bioabsorbable stents.

**Fig. 1:**
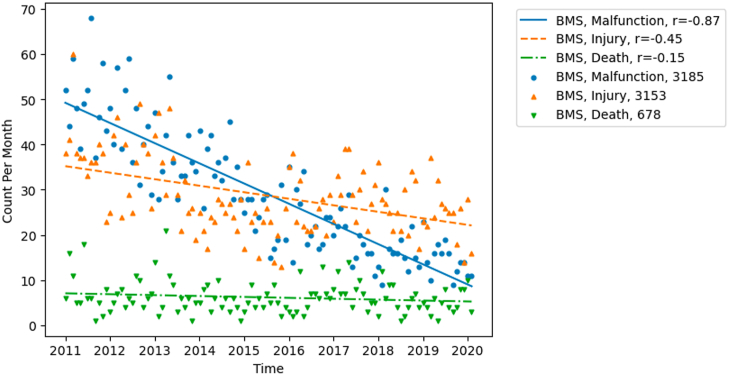
Trends in reported adverse events for BMS in FDA MAUDE. This figure demonstrates the data average by month, line of best fit, and corresponding R value of each device outcome category. BMS bare metal stents. FDA the U.S. Food and Drug Administration. MAUDE Manufacturer and User Facility Device Experience database.

**Fig. 2:**
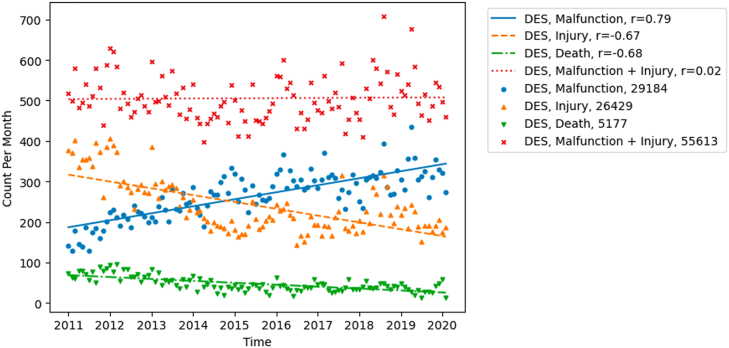
Trends in reported adverse events for DES in FDA MAUDE. The patient outcome Death, injury, and malfunction reports. Injury reports and malfunction reports compared to the summation of injury and malfunction reports. DES drug-eluting stents. FDA the U.S. Food and Drug Administration. MAUDE Manufacturer and User Facility Device Experience database.

Our analysis revealed steady decreases in the malfunctions and injuries of BMS between 2011 and 2020 (Fig. 1). In comparison, the noticeable increase for DES malfunctions was paired with a decrease in injuries during the study period (Fig. 2). Rates of death in both BMS and DES slightly decreased over time. For BVS, our analysis suggested a peak of all cases around 2017 with no clear increases or decreases. The equation representing the linear trend for malfunctions associated with BMS is y = −0.38x+51.7 with an R-value of −0.87. For DES, the equation for malfunctions is y = 1.5x+190.1 with an R-value of 0.79. For each equation each unit of x is one month while y is one reported case.

**Fig. 3:**
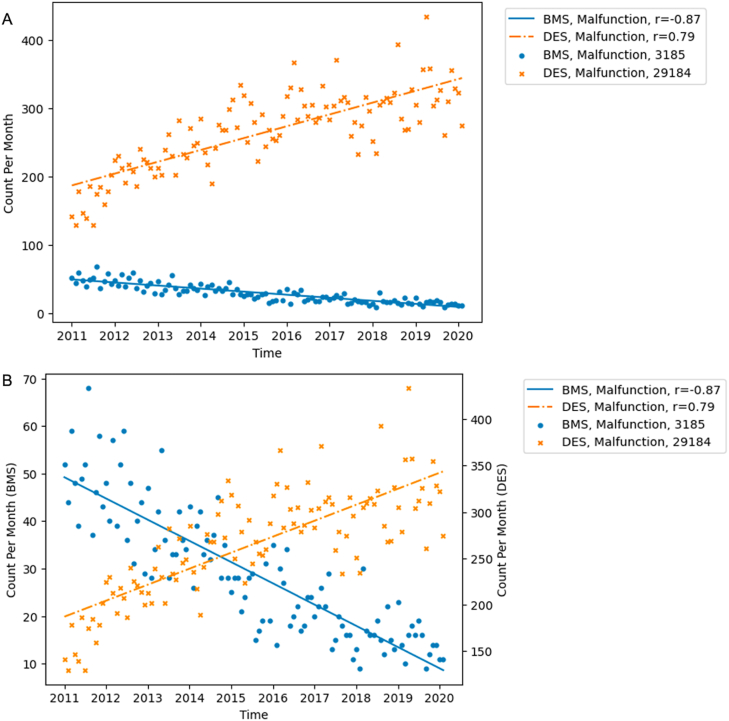
Comparison between DES and BMS for reported malfunctions in FDA MAUDE. (A) Single scale to show absolute trends. (B) Two scales to show relative trends. DES drug-eluting stents. BMS bare metal stents. FDA the U.S. Food and Drug Administration. MAUDE Manufacturer and User Facility Device Experience database.

When comparing the increase in injuries and decrease in malfunctions of DES over the study period, an inversely proportional change of the same magnitude was seen. As well, the sum of reports for injuries and malfunctions remained consistent over time (Fig. 2). When comparing the device outcome between different products, a similar trend was seen between BMS and DES. The slope-to-intercept ratio between the BMS and DES was of the same magnitude but reverse directionality, suggesting that the decrease in BMS malfunction rate is proportional to the increase in malfunctions of DES (Fig. 3). Comparing the raw averages, the increase in DES malfunction rate was, on average, 4 times the decrease seen in BMS.

When calculating the misreporting outcomes, BVS was not reported given the number of cases were relatively negligible (Table 3). In summary, 7.0 % of all reports were misreported, of which BMS represented 29.4 % while DES represented 70.3 %.

**Table 3:** Total count and misreported count of event narratives containing key death search terms. Total count includes all event narratives classified as deaths, injuries, or malfunctions. Misreported count includes all event narratives classified as injuries or malfunctions. Only BMS and DES reports were included in the analysis.

Year	Combined DES and BMS			BMS			DES		
	Misreport	Total	%	Misreport	Total	%	Misreport	Total	%
2011	38	616	6.17	3	62	4.84	35	554	5.96
2012	49	676	7.25	3	58	5.17	46	618	7.44
2013	35	515	6.80	3	53	5.66	32	462	6.93
2014	20	435	4.60	5	46	10.87	15	389	3.86
2015	18	372	4.84	4	37	10.81	14	334	4.19
2016	20	456	4.39	4	52	7.69	16	382	4.19
2017	34	405	8.40	22	76	28.95	11	315	3.49
2018	38	376	10.11	24	71	33.80	14	303	4.62
2019	41	329	12.46	18	58	31.03	23	270	8.52
Total	**293**	**4180**	**7.01**	**86**	**513**	**16.76**	**206**	**3627**	**5.68**

∗ BMS bare metal stents; DES drug eluting stents.

## 4 Discussion

Our results showed that between 2011 and 2020, there were decreased BMS and increased DES. Furthermore, an inversely proportional change of the same magnitude between injury and malfunction was demonstrated in DES. Finally, approximately 7 % of injury or malfunction device outcome reports were classified as misreports and may be more appropriately classified as death. These findings may allude to factors contributing to the choice of stents for angioplasty and changes to the approach of the MAUDE database maintenance, thus warranting further discussion.

Importantly, this study showed an inversely proportional change of different magnitude at 1:4 (BMS: DES) with decreasing BMS and increasing DES malfunctions. This trend was not clearly seen in the injuries and death outcome reports. One possible justification for the visualized trend is a transition from using BMS to DES, specifically second-generation DES, which was prominent between 2011 and 2020 [4,15]. Prior studies have suggested that this shift occurred because of the improved perioperative benefits in DES compared to BMS [16,17]. For example, a 2010 clinical trial suggested similar mortality but reduced revascularized rate in DES compared to BMS [18]. The advancements in the DES design, such as thinner struts and more biocompatible polymers, are attributable to these improved outcomes [19]. Additionally, second-generation DES exhibit a better safety profile and are associated with lower rates of stent thrombosis and myocardial infarction compared to BMS [20]. However, the increase in coronary disease population between 2011 and 2020 could not proportionally account for increasing malfunction rates in DES. Recent literature suggest a growing consensus that drug-eluting stents have lower rates of malfunction compared to bare-metal stents [7,21]. Therefore, it can be reasonably assumed that there is another underlying mechanism causing the steady increase in DES malfunctions, warranting further research.

Our results also revealed an inversely proportional change of the same magnitude between injury and malfunction rates of DES, suggesting that there could be a shift from injuries to malfunctions when reporting outcomes. A plausible explanation for this shift could be, as defined by MAUDE, that malfunctions are regarded as less severe than injuries. Indeed, the definition proposed by FDA suggested that malfunctions are described as an abnormal function of the device, while injuries are incidents where the device caused harm to the patient [22]. Given the inherent attraction towards reporting less severe cases, mandatory reporting facilities may opt to report an incident as malfunction instead of injury. As such, clearer criteria for device different outcome types and rigorous monitoring of these outcome reports may present as an effective strategy to promote accurate incident reporting.

Similar to existing literature, our results suggest the presence of misreporting in the MAUDE database, specifically at an approximate rate of 1 misreport per 14 reported cases [14]. This data suggests that companies are reporting device-related deaths as less severe outcomes such as injury or malfunction. A possible explanation for this finding is that loopholes exist in classifying device problems as a less severe outcome than experienced and can be used to the reporters’ advantage. A study on misreporting by Lalani et al. suggested a rate of 23 % for misreports [14]. One possibility of the discrepancy in the identified number of misreported records is the reporting key phrases may be different for coronary stent devices compared to the generalized key terms identified by Lalani et al. Therefore, the misreported cases are not identified through the searched key terms. Another possible reason is that given the standard for these minimally invasive procedures, there is less flexibility with the reporting of these devices [23]. However, constraints of software and design in this study offer opportunity for future studies to explore natural language processing to obtain a more accurate estimate of the misreporting rates for coronary stent devices. Furthermore, such technology can promote constant maintenance of discrepancies between reports and information to improve the accuracy of outcome reports in the MAUDE database.

Like all studies, this research has its limitations including the administrative nature of this database. Given the lack of details on the clinical trajectory of each case, inference on the causes of death, injury, and malfunctions cannot be interpreted. Moreover, all interpretations are limited to identifying trends and associations rather than establishing causal relationships. Furthermore, the timing at which each adverse event relative to the procedure performance time could not be analyzed. Additionally, the MAUDE database does not support cross-device comparison of absolute numbers of adverse event occurrence as suggested by FDA [11]. As well, while it is mandatory to report device cases from device companies, all sources of reporting from stakeholders such as physicians, nurses, or patients are under voluntary reporting [24]. Therefore, the MAUDE database does not rely on a comprehensive method of data collection. Given this limitation, specific data measures and values presented in the study should not be used for forecasting or projections. Majority of reports are obtained from the manufacturer. As such, variation in device selection and treatment strategies across practice from different physicians cannot be accounted within the analysis, limiting the granularity of interpreted trends. Together, the limitations serve as a guideline for further analysis of available data while providing direction for future research, given the clear correlation found between BMS and DES over a decade.

In conclusion, a 4-fold increase of DES to decrease of BMS in malfunction incidents was seen. Furthermore, a proportional increase of DES malfunction to decrease of DES injury incidence was visualized. These findings suggest that the stringency to reporting for the MAUDE database may be lacking in certain areas. Furthermore, BMS may be more favorable than previously expected compared to DES as a device-related outcome. With a lack of understanding in the underlying causes of the perceived risks and benefits of DES compared to BMS, future research should focus on understanding specific situations where BMS may be more favorable. As well, the blurred line between when to classify as injury or as malfunction for device reports implicate greater efforts to verify the accuracy and credibility of device outcomes classification within the MAUDE. As such, a better understanding and standardization of reporting criteria for device outcomes in the MAUDE database is needed.

## CRediT authorship contribution statement

**Zihan Gao:** Writing – review & editing, Writing – original draft, Visualization, Software, Project administration, Methodology, Formal analysis, Data curation, Conceptualization. **Willie Lei:** Validation, Software, Methodology, Formal analysis. **Eleanor Gao:** Writing – review & editing, Writing – original draft, Visualization. **Sujata Bhatia:** Writing – review & editing, Validation, Supervision, Project administration, Formal analysis, Data curation, Conceptualization.

## Ethics declaration

Given the data used for analysis was deidentified and accessible from a public domain, this study was exempted and did not require review and/or approval by an ethics committee or informed consent.

## Data availability statement

This data used the FDA Manufacturer and User Facility Device Experience (MAUDE) Database and is freely available to the public. The database can be accessed by following this link: https://www.accessdata.fda.gov/scripts/cdrh/cfdocs/cfmaude/search.cfm. Specific raw data presented in this study are included as supplementary materials. No data were deposited in any publicly available repositories. Further inquiries can be directed to the corresponding authors.

## Declaration of competing interest

The authors declare that they have no known competing financial interests or personal relationships that could have appeared to influence the work reported in this paper.
